# A Retrospective Analysis of the Clinicopathological Profile of Oral Squamous Cell Carcinoma in Tobacco and Non-tobacco Users: Highlighting the Significance of Chronic Mechanical Irritation

**DOI:** 10.7759/cureus.59953

**Published:** 2024-05-09

**Authors:** Priya Thomas, Deepu Mathew, Kutty Anisha, Abilasha Ramasubramanian, Karthikeyan Ramalingam, Pratibha Ramani, Durairaj Sekar

**Affiliations:** 1 Oral Pathology and Microbiology, Saveetha Dental College and Hospitals, Saveetha Institute of Medical and Technical Sciences, Saveetha University, Chennai, IND; 2 Oral and Maxillofacial Pathology and Oral Microbiology, Annoor Dental College & Hospital, Ernakulam, IND; 3 RNA Biology Lab, Saveetha Dental College and Hospitals, Saveetha Institute of Medical and Technical Sciences, Saveetha University, Chennai, IND

**Keywords:** younger individuals, chronic inflammation, non-habit, tobacco, trauma, chronic mucosal irritation, oral squamous cell carcinoma

## Abstract

Background and aim: Oral squamous cell carcinoma (OSCC) is among the leading top three cancers in India. However, recent literature has shown an increase in the rise of oral cancer in younger individuals without any history of tobacco-related habits. Chronic mucosal irritation (CMI) has been noted to have a substantial impact on the development and etiology of OSCC. With the shift in the trend, it is imperative to observe and monitor alterations associated with its etiological factors. The study aims to evaluate the prevalence and clinical characteristics of OSCC patients and the association of these parameters in cases with and without tobacco usage.

Methodology: A retrospective study spanning a period of 10 years was done on histopathologically diagnosed cases of OSCC. Various clinicopathological characteristics were collected from patient records, including demographic features, tobacco-related habits, including tobacco chewing and smoking, clinical presentation, anatomic sites, and histopathological grading based on the inclusion and exclusion criteria. The data were tabulated to Microsoft Excel (Microsoft Corporation, Redmond, WA), and descriptive statistics analysis and chi-square test of significance were applied to the data using IBM SPSS Statistics (version 29.0.2; IBM Corp., Armonk, NY). The study correlated the epidemiologic behavior of OSCC with age, gender, site, and tobacco-related habits.

Results: This study included a sample size of 204 (72 females & 132 males). Tobacco-related habit-associated cases were 98 (48.5%) and without tobacco habits were 61 cases (29.6%). Etiology associated with CMI emerged to be a significant tooth-related factor. Out of 72 females, 32 (44.4%) of the females were without habit. OSCC caused by trauma from CMI was analyzed in 40 cases (19.6%) and 22 (55%) were females. The majority of lesions (76 (37.4%) cases) presented on the lateral border of the tongue. Among the OSCC patients with a history of chronic mechanical irritation, 37 (48.7%) cases were observed to be specifically on the lateral border of the tongue.

Conclusion: These 10-year data will generate awareness about the disease pattern occurring within a community and provide an overview of the prerequisite of considering CMI as an etiological factor for the development of OSCC without the association of tobacco-related habits.

## Introduction

Oral squamous cell carcinoma (OSCC) is the most common cancer of the head and neck region in India, predominantly associated with tobacco usage. Recent literature has shown a rise in oral cancer in younger individuals without any history of tobacco-related habits. Chronic mechanical irritation (CMI) on the oral mucosa has been noted to have a substantial impact on the development and etiology of OSCC. With the shift in the trend, it is imperative to observe and monitor alterations associated with its etiological factors. The incidence of lip and oral cavity cancer in 2022, as reported by the Global Cancer Statistics (GLOBOCAN), ranking 16th globally, amounted to 389,846 cases, with an age-standardized rate (ASR) of 4.0. It ranked 15th in mortality, with 188,438 deaths and an ASR of 1.9. While cancer profiles partly mirror the significant incidence burdens of specific cancer types in densely populated countries, such as lung cancer in China and prostate cancer in the United States, the high prevalence of oral cavity cancer in India underscores a notable regional health concern [1]. According to predictions from the International Agency for Research on Cancer's GLOBOCAN project, the burden of oral cancer in India is anticipated to double in the next 20 years, exceeding more than 744,326 by 2040 [2].

GLOBOCAN data also reported 143,759 cases of lip and oral cancer in India, with the highest incidence rates in Melanesia and South-Central Asia (259,620 cases) in 2022. More than 90% of malignant oral tumors are squamous cell carcinoma [3]. In South Central Asia, OSCC represents a significant proportion of cancers, accounting for around 30% of all cancers, in contrast to developed countries where it comprises approximately 4% [4]. OSCC is among the top three cancers in India [5].

A notable shift in the patterns and trends of OSCC in developed countries in recent years, involving a shift in the distribution of fundamental risk factors, has been evident [6]. Traditional risk factors of OSCC are considered as tobacco usage (smoking, betel quid chewing, reverse smoking) either alone or in association with alcohol consumption. Other risk factors are related to lifestyle habits. Human papillomavirus (HPV) has been allied to oropharyngeal cancers (base of the tongue, lingual tonsil, and soft palate) in certain subpopulations of specific countries (men, younger ages, European racial origin). Additionally, lip cancers have been linked to ultraviolet radiation from sunlight exposure [7]. The alternate risk factors proposed include chronic irritation/trauma from ill-fitting dentures, rough, or fractured teeth, poor diet, low socioeconomic status, and poor hygiene [8].

However, recent studies and literature indicate a rising incidence of OSCC among younger individuals and women who are non-tobacco users [3]. Despite evolving epidemiological trends, oral cancer remains more prevalent among males, possibly due to increased exposure to risk factors. OSCC typically affects older age groups above the fifth decade, with a mean age of 62 years [9]. When considering the site of occurrence, tongue squamous cell carcinomas are found to be common, with an incidence varying between 25% and 40% [10].

Based on the current scenario, there is a high necessity to analyze the changes associated with the etiological factors of OSCC. Approximately 275,000 new cases of OSCC are identified each year globally, but reports have shown that the incidence of OSCC shows large geographical variations. Reviewing the literature depicted minimal data correlating the clinicopathological features related to the Indian continent, especially to the South Indian population related to Kerala. Considering the compound dynamics of underlying risk factors, different characteristics and trajectories in oral cancer, and the shift in epidemiologic patterns, the present retrospective study was performed to evaluate and analyze the clinicopathological features of OSCC and identify the drifts in the number of cases or incidence rates at precise anatomic sites or within specific ages or genders in the central Kerala population. We also sought to evaluate the strength of the association of the clinicopathologic characteristics in OSCC patients with and without tobacco usage.

## Materials and methods

The current retrospective study conducted at Annoor Dental College & Hospital, Kerala aimed to analyze the clinical data related to cases of OSCC. The ethical approval for the study was obtained from the Institutional Human Ethical Committee, Annoor Dental College & Hospital, Kerala (IHEC/022-B/34). Informed consent was waived due to the retrospective nature of the study, and the analysis utilized patient records; however, measures were taken to protect patient confidentiality.

The clinical data were obtained from the chart review of histopathologically diagnosed cases within the timeframe from January 2012 to September 2022, extracted from the archives of the Department of Oral Pathology of the Annoor Dental College, Kerala, India. The patient's history, demographic features like age and gender, tobacco-related and alcohol habits, clinical presentation of the lesion, anatomic sites or location of the lesion, duration of the lesion, and histopathological grading were the variables considered for the study. The pathophysiologic and epidemiologic behaviors of OSCC were correlated with age, gender, site, and habit.

Inclusion criteria included all histopathologically confirmed cases of OSCC diagnosed between January 2012 and September 2022, encompassing primary, recurrent, and metastatic cases. We considered patients of all demographic backgrounds, ensuring the availability of comprehensive clinical and pathological data for analysis, including demographic features (age, gender), clinical presentation (anatomic site, lesion characteristics), tobacco-related habits, alcohol consumption history, duration of the lesion, and histopathological grading. Cases specifically diagnosed as metastatic squamous cell carcinoma (SCC) from primary tumors located in different anatomical regions were also included. Exclusion criteria comprised non-OSCC malignancies within the oral cavity, cases of oral epithelial dysplasia without invasive SCC, metastatic lesions to the oral cavity originating from non-epithelial malignancies, and patients with a history of non-oral primary malignancies unless concurrently diagnosed with OSCC.

All the data retrieved were tabulated in Microsoft Excel (Microsoft Corporation, Redmond, WA), and descriptive statistical analysis was conducted for demographic and clinical variables. The male-to-female ratio, the mean age of occurrence of OSCC cases, the frequency of occurrence of OSCC with the anatomic locations, and various clinical presentations like non-healing ulcers, ulceroproliferative lesions, or white patches were documented. The association between OSCC and tobacco-related habits or trauma from chronic irritation was analyzed. The OSCC lesions were categorized based on their histopathological grading into well-differentiated OSCC (WDSCC), moderately differentiated OSCC (MDSCC), and poorly differentiated OSCC (PDSCC). The chi-square test was used for the assessment of the level of significance and to assess the associations between variables such as gender, age, site of occurrence, histopathological grading, and habits. A p-value of less than 0.05 was taken as significant.

## Results

Demographics

During the specified timeframes, a total of 204 patients diagnosed with OSCC were identified. Among these cases, 132 (64.7%) were male, while 72 (35.2%) were female, establishing a male-to-female ratio of 11:6. The mean age of OSCC cases was 60.5 years, ranging from 31 to 92 years. Predominantly, the fifth and sixth decades exhibited the highest susceptibility, encompassing 104 (50.9%) cases, followed by the seventh decade with 39 (19.1%) cases. Notably, 18 (8.8%) cases occurred in patients below 41 years of age. A total of 72 (68.05%) among females and 93/132 (70.45%) among males belonged to the 5th-7th decade (Table 1).

**Table 1:** Age and gender distribution among oral squamous cell carcinoma cases. The table displays oral squamous cell carcinoma (OSCC) cases by gender. Out of 204 cases, 132 (64.7%) were males and 72 (35%) were females. The mean age was 60.5 years, ranging from 31 to 92 years. The fifth and sixth decades had the highest susceptibility (104, 50.9%), followed by the seventh decade (39, 19.1%), with 18 (8.8%) cases below 41 years.

Age group (years)	Total number of cases, N (%)	Females, N (%)	Males, N (%)
31 - 40	18 (8.82%)	7 (3.4%)	11 (5.3%)
41 - 50	29 (14.21%)	8 (3.9%)	21 (10.29%)
51 - 60	57 (27.9%)	17 (8.33%)	40 (19.6%)
61 - 70	47 (23.04%)	15 (7.35%)	32 (15.7%)
71 - 80	39 (19.11%)	17 (8.33%)	22 (10.78%)
81 - 90	12 (5.88%)	8 (3.9%)	4 (1.96%)
91 - 100	1 (0.49%)	-	1 (0.49%)
Missing	1	-	1
Total	204	72 (35.29%)	132 (64.7%)

Distribution of males & females affected by OSCC

Our study illustrated a trend of increasing male cases affected by OSCC from 2012 to 2022 and females from 2019 to 2022. While there were fluctuations in the number of cases across the years, notable peaks in males were observed in 2015, 2018, 2021, and 2022. These peaks may have indicated periods of heightened incidence, improved detection rates, or variations in risk factors influencing OSCC development (Table 2).

**Table 2:** Distribution of males and females affected by oral squamous cell carcinoma (OSCC) over the period from 2012 to 2022. Notably, there is an increasing trend observed from 2012 to 2022, with a gradual rise in the number of affected males and females. There are noticeable peaks of males in 2015, 2018, 2020, 2021, and 2022, suggesting potential periods of heightened incidence or improved detection rates.

S. No.	Year	No. of males affected with OSCC	No. of females affected with OSCC
1	2012	7	4
2	2013	2	3
3	2014	8	4
4	2015	17	1
5	2016	4	1
6	2017	9	2
7	2018	15	5
8	2019	13	14
9	2020	15	10
10	2021	19	15
11	2022	23	13

Site of occurrence

**Figure 1:**
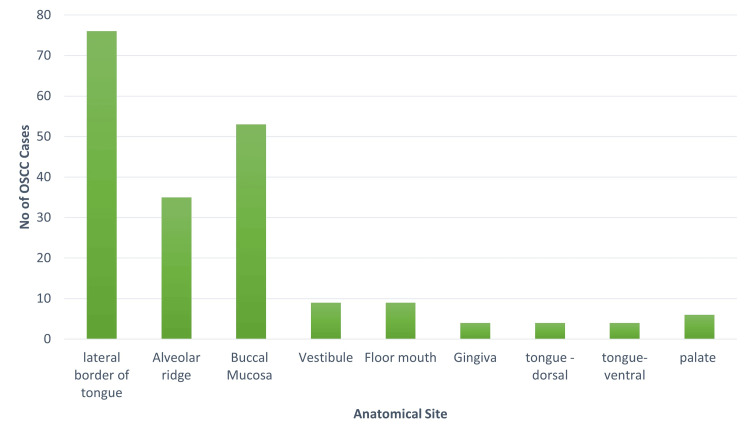
Graph representing the site predilection for oral squamous cell carcinoma. The graph illustrates the distribution of oral squamous cell carcinoma (OSCC) cases across different anatomical sites. The lateral border of the tongue shows a concentration of cases compared to other sites.

The lateral border of the tongue was identified as the most frequent site of OSCC occurrence, accounting for 76 (37.4%) cases, followed by the buccal mucosa with 53 (25.9%) cases, and occurrences in the alveolar mucosa and retromolar area. Additional occurrences were noted in the gingiva, the floor of the mouth, vestibule, dorsal and ventral surfaces of the tongue, hard palate, and notably, one case in the esophagus (Figure 1).

Duration and clinical presentation

Most patients reported durations ranging from one to six months, from the onset of symptoms to the time of biopsy, with fewer cases exceeding 12 months (14 cases). Notably, three cases exhibited extended durations of 120, 158, and 168 months, respectively. Non-healing ulcers or ulcerated lesions were the most common clinical presentation observed in 85 cases (41.6%), followed by ulceroproliferative masses in 38 cases (18.6%), leukoplakic or white patches in 21 cases (10.3%), and leukoerythroplakia (speckled lesions) in 15 cases (7.4%). Other presentations included proliferative/papillary/fungating growths and swelling with pain. Additionally, two cases originated from unhealed extraction sockets, while two patients had a history of potentially malignant disorders, specifically oral submucous fibrosis.

Tobacco-related habit association, trauma, and age

Among the 204 OSCC cases, 98 (48.5%) were associated with tobacco-related habits, while 60 (29.6%) had no habits, and data were unavailable for 45 (21.8%) cases (Table 3).

**Table 3:** Descriptive statistics for the association between age and tobacco-related habit/without tobacco-related habit. Analysis reveals that 98 (48.5%) cases had tobacco-related habits, 60 (29.6%) without habits, and 45 (21.8%) cases had no data. Among the majority age group (51-60 years, 58 cases), 27 (48.5% cases) had tobacco habits, while 17 (29.3%) cases did not. Pearson chi-square test yielded a p-value of 0.106, indicating nonsignificant differences in tobacco habit prevalence across age groups.

Age group (in years)	Total no. of cases (N)	Tobacco-related habits, N (%)	No habits, N (%)	Data insufficient, N (%)	P-value (Pearson chi-square)
31 - 40	18	8 (44.4%)	10 (55.6%)	0	0.106
41 - 50	30	14 (46.6%)	11 (36.6%)	5 (16.6%)	
51 - 60	58	27 (46.5%)	17 (29.3%)	14 (24.1%)	
61 - 70	45	24 (53.3%)	10 (22.2%)	11 (24.3%)	
71 - 80	39	18 (46.2%)	8 (20.5%)	13 (33.3%)	
81 - 90	12	7 (58.3%)	4 (33.3%)	1 (8.3%)	
91 - 100	1	0	0	1 (100.0%)	
Total	203 (100.0%)	98 (48.5%)	60 (29.7%)	45 (21.8%)	

Among the 98 habit-associated cases, only six reported both trauma from chronic irritation (either sharp tooth/fixed partial denture/ill-fitting denture) and tobacco-related habits. A significant correlation was observed between tobacco-related habit association and gender (p = 0.005), with OSCC more prevalent in males (70/132 cases; 53.0%) compared to females (28/72 cases; 38.9%) (Table 4).

**Table 4:** Gender-wise distribution of OSCC cases with tobacco-related habits and trauma from chronic mechanical irritation. Gender-wise distribution of oral squamous cell carcinoma (OSCC) cases with tobacco-related habits and trauma from chronic mechanical irritation (CMI) reveals a significant correlation: 65 males had tobacco-related habits associated with OSCC, compared to 27 females. Additionally, 22 females and 18 males reported a history of trauma stemming from CMI. Notably, 21 cases did not exhibit any association with either trauma or habits.

Gender	Tobacco-related habit	No habit	Trauma	Data missing	Trauma + habit	Total cases
Male	65	11	18	33	5	132
Female	27	10	22	12	1	72
Total	92	21	40	45	6	204

**Figure 2:**
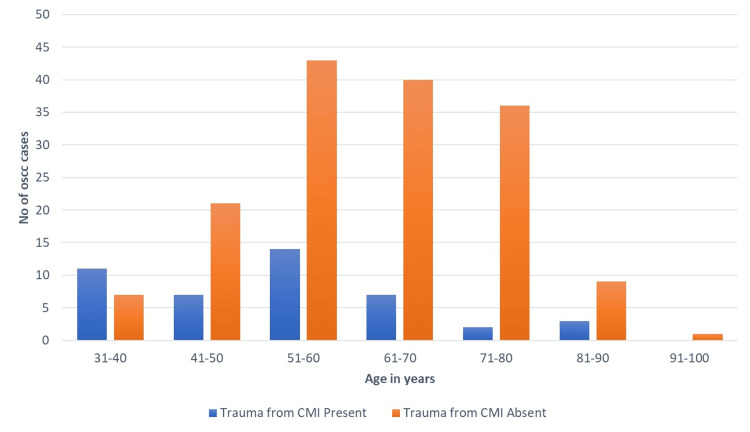
Graph representing the association of age and trauma from chronic mechanical irritation. This graph illustrates the relationship between age and cases of chronic mechanical irritation (CMI) trauma leading to oral squamous cell carcinoma (OSCC). The highest number of cases occurs between the age of 50 and 60 years.

Among the documented cases, 21 did not present associations with trauma from CMI or tobacco habits, indicating the absence of these two identified risk factors. Trauma or chronic irritation was identified as a causative factor in 40 cases (19.6%), with 22 (10.8%) cases in females. There was also a notable association between age and trauma (p = 0.000), peaking in the 51-60 years age group, followed by the 31-40 years age group (Figure 2).

Etiological factors associated with OSCC

Notably, there was a noticeable increase in cases attributed to sharp cusps or tooth-related trauma from 2017 onwards, peaking in 2022. Additionally, the incidence of OSCC cases associated with ill-fitting dentures showed fluctuations, with a notable increase in 2020 and subsequent years. The combined influence of habits (tobacco-related) with trauma, particularly from ill-fitting dentures, was also recorded (Table 5).

**Table 5:** Etiological factors associated with OSCC. Notably, there is a noticeable increase in cases attributed to sharp cusps or tooth-related trauma from 2017 onwards, peaking in 2022. Additionally, the incidence of OSCC cases associated with ill-fitting dentures has shown fluctuations, with a notable increase in 2020 and subsequent years. The combined influence of habits (tobacco-related) with trauma, particularly from ill-fitting dentures, has also been recorded in the latter years of the dataset. OSCC: oral squamous cell carcinoma; FPD: fixed partial denture.

S. No.	Year	Sharp cusp/tooth	FPD	Ill-fitting denture	Tobacco-related habit + sharp tooth	Tobacco-related habit + trauma from ill-fitting denture
1	2012	2				
2	2013	0				
3	2014	0				
4	2015	0				
5	2016	1				
6	2017	1				
7	2018	2				
8	2019	5				
9	2020	8	1	1	1	
10	2021	9			2	
11	2022	12			2	1
	Total	38			5	

Site and tobacco-related habit relation profiling

A compelling association was observed between the site of OSCC occurrence and habit relation. The lateral border of the tongue exhibited a higher number of cases associated with trauma from mechanical irritation (37 cases) compared to cases associated with tobacco-related habits, which was statistically significant (p < 0.000). Thirty-three cases in the buccal mucosa occurred in association with tobacco-related habits. Non-habit-associated cases, not specified in the above categories, were observed in the lateral border of the tongue (five cases), alveolar ridge, buccal mucosa, ventral and dorsal surfaces of the tongue, palate, and gingiva (Table 6).

**Table 6:** Association between site and trauma caused by CMI from sharp tooth or prosthesis. A significant correlation was found between the oral squamous cell carcinoma (OSCC) site and habit association. The lateral tongue border exhibited a higher incidence of cases associated with chronic mechanical irritation (CMI; 37 cases) compared to tobacco-related habits, which was statistically significant (p = 0.000, Pearson chi-square test).

Site	Total no. of cases (N)	Trauma from CMI		P-value (Pearson chi-square)
		Present, N (% within site)	Absent, N (% within site)	
Lateral border tongue	76	37 (48.7%)	39 (51.3%)	0.000
Alveolar ridge	35	2 (5.7%)	33 (94.3%)	
Vestibule	9	0	9 (100.0%)	
Floor mouth	9	0	9 (100.0%)	
Tongue ventral	4	2 (50.0%)	2 (50.0%)	
Dorsum of tongue	4	1 (25.0%)	3 (75.0%)	
Palate	6	0	6 (100.0%)	
Gingiva	4	1 (25.0%)	3 (75.0%)	
Angle of mouth	2	0	2 (100.0%)	
Esophagus	1	0	1 (100.0%)	
Buccal mucosa	53	3 (5.7%)	50 (94.3%)	
Total	203	46 (22.7%)	157 (77.3%)	

Histological grades

**Figure 3:**
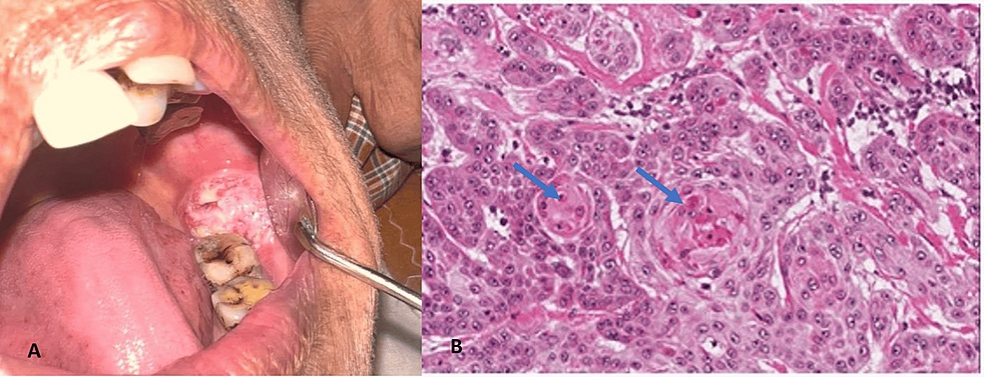
(A) Clinical appearance of ulceroproliferative growth present on the retromolar pad. (B) Histopathology of well-differentiated oral squamous cell carcinoma (hematoxylin & eosin stain, 40x). A: Clinical appearance of ulceroproliferative growth present since three months on the retromolar pad. B: Histopathology of well-differentiated oral squamous cell carcinoma (hematoxylin & eosin stain, 40x) with islands of malignant tumor cells showing attempting keratin pearl formation (blue arrow).

**Figure 4:**
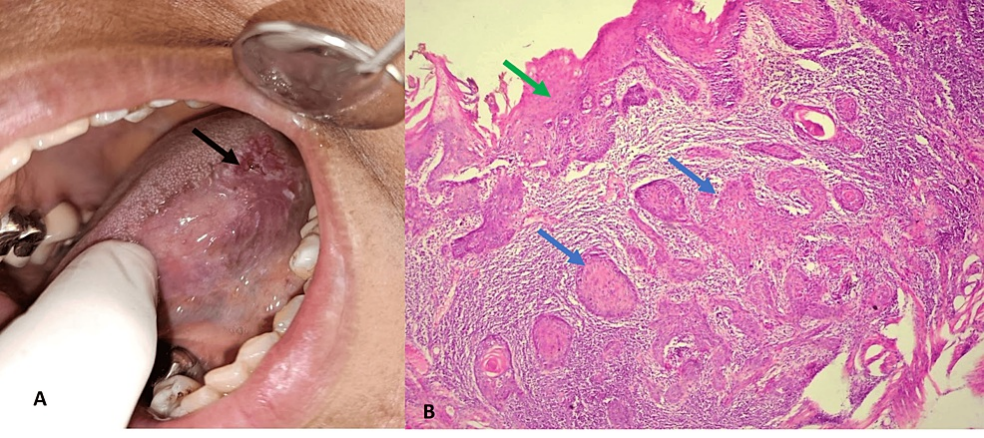
(A) Clinical appearance of non-healing ulcer on the left lateral border of the tongue. (B) Histopathology of moderately differentiated oral squamous cell carcinoma (hematoxylin & eosin stain, 10x). A: Clinical appearance of non-healing ulcer on the left lateral border tongue (black arrow) extending to the ventral part of the tongue in relation to 36. B: Histopathology of moderately differentiated oral squamous cell carcinoma (hematoxylin & eosin stain, 10x) with islands and sheets of malignant tumor cells (blue arrow) in the connective tissue and overlying dysplastic oral epithelium (green arrow).

**Figure 5:**
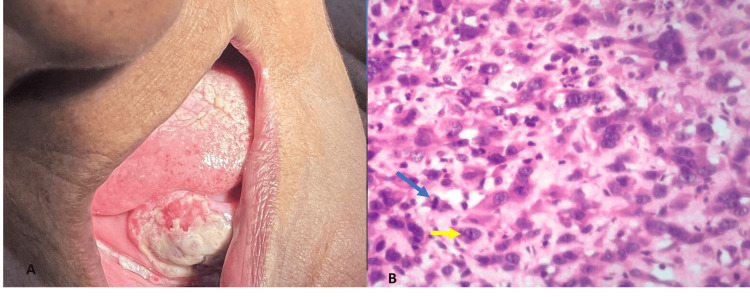
(A) Ulceroproliferative growth on the right alveolar ridge. (B) Histopathology of poorly differentiated oral squamous cell carcinoma (hematoxylin & eosin stain, 40x). A: Ulceroproliferative growth on the right alveolar ridge. B: Histopathology of poorly differentiated oral squamous cell carcinoma (hematoxylin & eosin stain, 40x) with pleomorphic tumor cells exhibiting increased nuclear-cytoplasmic ratio, prominent nucleoli (yellow arrow), and mitotic figure (blue arrow).

Histopathological grading of OSCC revealed WDSCC in 66 cases (32.35%) (Figure 3), MDSCC (Figure 4) in 73 cases (35.78%), and PDSCC in 18 cases (8.3%) (Figure 5).​​

Additionally, diagnosis of superficially invasive carcinoma or microinvasive carcinoma (29 cases) and OSCC (10 cases) were reported. Microscopic variants included four cases of verrucous carcinoma and one case of spindle cell carcinoma.

Recurrent & metastatic cases and previous history

Two patients exhibited prior carcinoma history: one with lung SCC diagnosed five years earlier, presenting with metastasis to the gingiva, and the other with verrucous carcinoma on the tongue in 2017, which progressed to OSCC within four years. Furthermore, a metachronous tumor emerged over a four-year period, with the primary lesion on the palate and the secondary lesion on the tongue. Additionally, two cases were categorized as recurrent, with one patient having undergone radiation therapy seven years prior and another demonstrating familial predisposition.

Associations between age, gender, and histopathological grading

No significant association was found between age, gender, and histopathological grading of OSCC. However, concerning site and grading, the lateral border of the tongue presented 32 out of 77 cases of MDSCC, while the buccal mucosa demonstrated 23 out of 53 cases of WDSCC.

## Discussion

Nearly 90% of oral malignancies originate from epithelial cells [11]. India, recognized as a global hub for cancers, particularly faces a significant burden of oral cancer, notably affecting males with an annual incidence of 45,455 and a mortality rate of 31,102. India ranks as the fourth highest country in terms of oral cancer incidence among males and the fifth highest among females, highlighting a major health concern [12,13]. Individuals from lower socio-economic backgrounds are disproportionately affected due to lifestyle risk factors, although recent literature suggests a shifting pattern toward the occurrence of OSCC from CMI. Despite a wealth of global research on OSCC, studies specific to India, especially in the South Indian population like Kerala, are limited. Thus, our retrospective study aimed to evaluate OSCC clinicopathological features and identify trends in case numbers and incidence rates at specific anatomic sites, ages, and genders within central Kerala.

In the Indian context, the age-adjusted rates of oral cancer exceed 20 per 100,000 population [12]. Notably, OSCC presents more aggressively in younger age groups compared to older individuals [14], with its highest incidence typically occurring during the sixth and seventh decades of life. While only a small percentage of oral cancer cases manifest in individuals under 40 years old (0.4% to 3.6%) [15], our study documented patients as young as 31 years old, with 12.25% below the age of 45, indicating a slight increase compared to previous reports [16,17].

Regarding age distribution, a significant proportion of our patients were in their fifth decade, aligning with existing Indian and global literature [18,19]. This observation underscores age as a key factor in OSCC manifestation.

Various forms of tobacco, including betel quid, tobacco with lime, bidi, and hookah, pose substantial risk factors, particularly in Southeast Asia [3]. Chronic and heavy tobacco use, combined with alcohol consumption, significantly increases OSCC risk, with a notable male preponderance attributed to escalating tobacco usage [3]. In the existing study, only a minority (7.1%) among the habit-related cases used both these substances. Also, the concurrent utilization of chewing and smoking tobacco compounds the risk of cancer onset. The results affirm the enduring significance of tobacco as the primary causative agent.

Chronic inflammation has been considered the seventh hallmark of cancer. Among the less-explored risk factors is CMI. Many reports have been illustrated in the literature pertaining to OSCC with a history of trauma either from tooth-related factors like poor dentition, sharp cusps/malocclusion [20,21], missing teeth [22], and prosthesis or ill-fitting dentures with sharp edges [23]. Singhvi et al. have established a robust association between ill-fitting dentures and OSCC [24]. In our analysis, trauma-related OSCC cases frequently reported CMI from sharp dental cusp or denture irritation with 38/40 cases specifically attributed to sharp cusp and two cases to denture irritation.

Notably, chronic irritation, compounded by tobacco exposure, exacerbates inflammation, contributing to OSCC pathogenesis [23,25]. This underscores the intricate interplay between mechanical irritation, tobacco habits, and their potential synergistic effects in the pathogenesis of oral malignancies. The findings accentuate the multifactorial etiology of OSCC and contribute to an innate perception of the implicated factors in its development. Time-dependent correlations between CMI and OSCC further emphasize the importance of early intervention [21]. CMI by itself may not directly produce genetic mutations but may accelerate epigenetic changes that ultimately impede DNA repair and apoptosis. Animal experimental models have depicted that malignancy could arise in a field, if chronic traumatic ulcer produced by constant irritation from tooth sharp ends or restorations, is triggered by carcinogens viz tobacco or alcohol [26]. This could be explained by the five cases in our data wherein chronic irritation was associated with tobacco.

Anatomically, OSCC exhibits site-specific preferences, with the tongue's lateral border emerging as a common and aggressive subsite associated with higher mortality [27-29]. Interestingly, our findings diverged from previous studies, highlighting the lateral border of the tongue as a predominant site, often linked to dental trauma [19,30]. A total of 38 cases documented in our study were associated with chronic irritation from sharp teeth. This underscores the need for further research to elucidate the mechanisms driving OSCC development at specific subsites, particularly among younger individuals.

Commonly known risk factors, such as tobacco, were observed in most of our cases; however, the increasing trend of OSCC among younger groups, especially at aggressive subsites like the lateral border of the tongue, underscores the urgency for targeted preventive strategies. OSCC affecting the tongue represents an aggressive form, characterized by a heightened proclivity toward invasion and metastasis. Comprehensive patient histories, including family history, are essential for understanding evolving OSCC profiles.

Limitations of our study include its single-institutional scope and reliance on retrospective clinical data, which may limit generalizability. Future studies should aim for broader geographic coverage and prospective data collection to enhance our understanding of OSCC trends and risk factors.

## Conclusions

This retrospective study sheds light on the clinicopathological characteristics and evolving trends of OSCC within central Kerala, India. Key findings underscore the predominance of OSCC among males, with an increasing incidence observed in younger age groups and individuals without traditional risk habits. While tobacco use and alcohol consumption remain significant contributors to OSCC, the emergence of cases in younger patients suggests a shifting epidemiological landscape that warrants targeted preventive measures.

Specific site preferences within the oral cavity, notably the lateral border of the tongue, highlight the impact of chronic dental trauma and mechanical irritation as potential etiological factors. This study emphasizes the need for comprehensive surveillance strategies and multidisciplinary approaches to address OSCC's evolving patterns, especially among vulnerable populations. Moving forward, efforts should prioritize public health education, early detection through screening initiatives, and interventions tailored to the changing risk profiles observed in regions like Kerala, India, to combat the burden of this prevalent oral malignancy effectively. Future research should focus on broader geographic representation and prospective data collection to further refine our understanding and enhance preventive strategies against OSCC.
